# An integrated meta-omics approach reveals substrates involved in synergistic interactions in a bisphenol A (BPA)-degrading microbial community

**DOI:** 10.1186/s40168-019-0634-5

**Published:** 2019-02-06

**Authors:** Ke Yu, Shan Yi, Bing Li, Feng Guo, Xingxing Peng, Zhiping Wang, Yang Wu, Lisa Alvarez-Cohen, Tong Zhang

**Affiliations:** 1 School of Environment and Energy, Shenzhen Graduate School, Peking University, Shenzhen, China; 2 Environmental Biotechnology Laboratory, The University of Hong Kong, Pokfulam road, Hong Kong, China; 3 Department of Civil and Environmental Engineering, University of California at Berkeley, Berkeley, USA; 4 Guangdong Provincial Engineering Research Center for Urban Water Recycling and Environmental Safety, Graduate School at Shenzhen, Tsinghua University, Shenzhen, China; 5 School of Life Sciences, Xiamen University, Xiamen, China; 6 School of Environmental Science and Engineering, Sun Yat-sen University, Guangzhou, China; 7 School of Environmental Science and Engineering, Shanghai Jiao Tong University, Shanghai, China; 8 Earth Science Division, Lawrence Berkeley National Laboratory, Berkeley, California USA; 9 Environmental microbiology and bioinformatics Laboratory, Shenzhen Graduate School, Peking University, Nanshan district, Shenzhen, Guangdong China

**Keywords:** Integrated meta-omics, Bisphenol A, Bacterial interactions, Biodegradation

## Abstract

**Background:**

Understanding microbial interactions in engineering bioprocesses is important to enhance and optimize performance outcomes and requires dissection of the multi-layer complexities of microbial communities. However, unraveling microbial interactions as well as substrates involved in complex microbial communities is a challenging task. Here, we demonstrate an integrated approach of metagenomics, metatranscriptomics, and targeted metabolite analysis to identify the substrates involved in interspecies interactions from a potential cross-feeding model community—bisphenol A (BPA)-biodegrading community, aiming to establish an identification method of microbial interactions in engineering or environmental bioprocesses.

**Results:**

The community-level BPA-metabolic pathway was constructed using integrated metagenomics and targeted metabolite analyses. The dynamics of active functions and metabolism of major community members were identified using metagenomic and metatranscriptomic analyses in concert. Correlating the community BPA biodegradation performance to the individual bacterial activities enabled the discovery of substrates involved in a synergistic interaction of cross-feeding between BPA-degrading *Sphingonomas* species and intermediate users, *Pseudomonas* sp. and *Pusillimonas* sp. This proposed synergistic interaction was confirmed by the co-culture of a *Sphingonomas* sp. and *Pseudomonas* sp. isolates, which demonstrated enhanced BPA biodegradation compared to the isolate of *Sphingonomas* sp. alone.

**Conclusion:**

The three types of integrated meta-omics analyses effectively revealed the metabolic capability at both community-wide and individual bacterial levels. The correlation between these two levels revealed the hidden connection between apparent overall community performance and the contributions of individual community members and their interactions in a BPA-degrading microbial community. In addition, we demonstrated that using integrated multi-omics in conjunction with culture-based confirmation approach is effective to elucidate the microbial interactions affecting the performance outcome. We foresee this approach would contribute the future application and operation of environmental bioprocesses on a knowledge-based control.

**Electronic supplementary material:**

The online version of this article (10.1186/s40168-019-0634-5) contains supplementary material, which is available to authorized users.

## Background

Environmental engineering bioprocesses, such as bioremediation and biological wastewater treatment, rely on the collective activities of mixed microbial populations to achieve desirable performance outcomes [1]. The microbes in these bioprocesses often compete or collaborate with each other to utilize the available chemicals [2–4]. For instance, *Rhodococcus rhodochrous* S-2 produced extracellular polysaccharides, containing nutrients and aromatic fractions. These products resulted in the emulsification of aromatic fractions, promotion of the growth of indigenous bacteria, e.g., *Cycloclasticus* spp., and enhancement of the degradation of aromatic fraction by the bacteria [5]. A *Rhodanobacter* strain that was unable to grow on benzo[a]pyrene in pure culture grew on metabolites produced by other consortium members and strongly contributed to benzo[a]pyrene mineralization by increasing its bioavailability [6]. *Dehalococcoides mccartyi* consumes hydrogen and chlorinated ethenes to maintain the exergonic and non-inhibitory states for non-dechlorinating fermenters which in turn provide hydrogen, acetate, and essential nutrients while removing inhibitory byproducts from *D. mccartyi* [3, 7–12]. The understanding of these microbial interactions has been crucial in developing not only strategies to boost bioremediation performance but also tools to monitor and predict the success of the bioprocesses.

However, unraveling microbial interactions in complex microbial communities is a challenging task, which requires thorough identification of major microorganisms and their individual physical and metabolic capabilities and states in the context of overall community-level performances. Because of the ability to provide molecular details at different complexity levels, metagenomics, metatranscriptomics, and metabolomics are promising to investigate the microbial interactions. While metagenomics reveals the functional potential, metatranscriptomics and metabolomics uncover active genes and metabolic responses to specific physiological processes in complex microbial communities [13–18]. Since different types of meta-omics analyses can complement and mutually support each other, integrated meta-omics datasets can yield more in-depth and thorough understanding of microbial communities beyond the totality of each individual dataset. As a result, patterns of the co-occurrence and activity correlation emerge from different microbial groups within communities [19, 20]. Currently, the integrative analysis of various meta-omics data is still limited and has not yet been employed to study bisphenol A (BPA)-degrading microbial communities [21].

BPA is a heavily produced chemical monomer that has been widely used in food package coating and synthesis of polycarbonate plastics and epoxy resins [22, 23]. If released into the environment, BPA, an endocrine disruptor, can cause adverse effects to ecology and public health [24, 25]. Although BPA does not persist significantly under aerobic conditions, incomplete degradation of BPA has been reported during wastewater treatment and imposes threats to the aquatic environments receiving treated effluent [26–29]. Therefore, strategies that promote the fast and complete BPA degradation are important for the wastewater treatment.

A number of bacterial isolates have demonstrated the capability of mineralizing BPA, including *Sphingomonas* sp., *Acromobacter xylosoxidans*, *Cupriavidus basilensis*, and *Bacillus pumilus* [23, 30, 31]. Interestingly, faster mineralization rates have generally been reported for microbial communities than for BPA-degrading isolates. A co-culture of BPA-degrading and non-degrading bacterium demonstrated faster rates than the culture of BPA-degrading bacterium alone, indicating that unknown microbial interactions likely expedite complete BPA biodegradation [32]. To identify the microbial interactions that support the efficient BPA biodegradation, this study developed an analytical pipeline of integrated meta-omics to dissect the metabolic capabilities and interactions of microbial members in a BPA-degrading community. These analyses showed a hypothetical substrate cross-feeding between BPA-degrading and non-degrading populations in the microbial community. Culture-dependent methods were then applied to validate the performance-enhancing interactions identified from integrated multi-omics analysis.

## Results

### BPA-degrading microbial community and meta-omics analysis pipeline

**Fig. 1:**
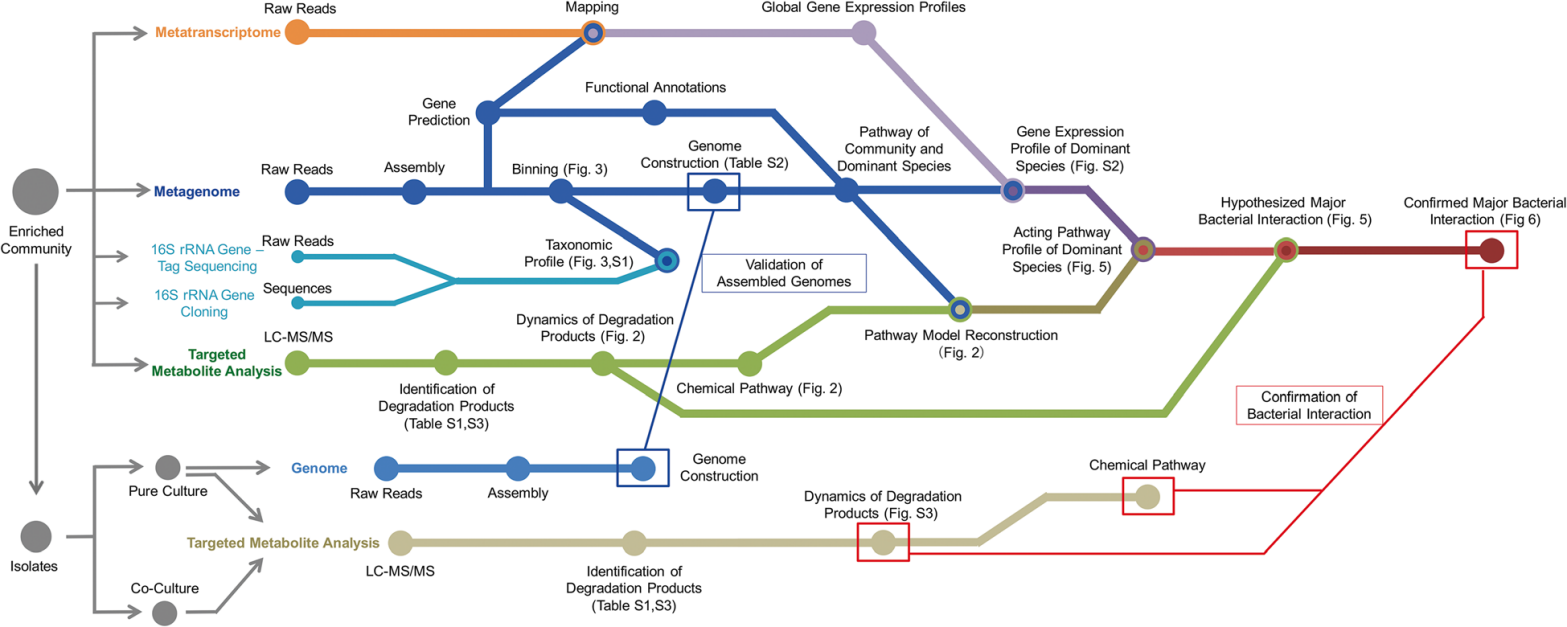
Scheme of the experimental design and analytical pipeline used in this study. Targeted metabolite analysis (green lines) was used to investigate the biodegradation intermediates and dynamics, which were integrated with metagenomic annotation (blue lines) to construct the community-wide BPA-mineralizing pathways. Integrated analyses of 16S-sequencing (light blue lines), metagenomics, and metatranscriptomics (orange lines) identified functionally active populations and metabolic pathways of individual strains in the community. Correlation of individual activities and overall community-wide BPA mineralization revealed the interactions of major community populations (thick red line) which was confirmed by the genomic and metabolite analysis of bacterial isolates from the community (fine blue line and fine red line, respectively)

We enriched a BPA-degrading culture from activated sludge using BPA as the sole electron donor and carbon source. BPA concentrations were increased from 20 to 50 mg L^−1^ over a 5-month period. A workflow was developed for analyzing and integrating various meta-omics datasets in order to investigate the metabolic capabilities and correlations between different bacterial populations in the BPA-degrading community. This workflow was also used to integrate pure culture isolation and analysis to test the proposed metabolic interaction model revealed from meta-omics analyses (Fig. 1).

We used three major types of integrated analysis to identify differences in encoded and expressed microbial functions in the context of metabolite variation in the BPA-degrading microbial communities. First, since many transient intermediates were not detected by liquid chromatography in conjunction with tandem mass spectrometry (LC-MS/MS), we integrated the metabolic capacity identified by functional annotation of metagenomic data to the metabolite analysis to reconstruct the community-wide BPA-mineralizing pathway. Second, we combined the metabolic capabilities identified for each microbial genome from the metagenomics data with gene expression profiles obtained from the metatranscriptomics to obtain the gene expression of specific dominant pathways. 16S rRNA gene analysis confirmed the dominance of the bacterial populations identified from metagenomic data. Mapping of dominant gene expression to the community-wide BPA-mineralizing pathway thus revealed the pathway profile of dominant species and the potential roles of individual species to the overall degradation. Finally, we isolated dominant strains from the enrichment to confirm the metabolic interactions identified from meta-omics analyses.

### Reconstruction of community-level BPA-mineralizing pathway using metabolite analysis and functional annotation of metagenome

**Fig. 2:**
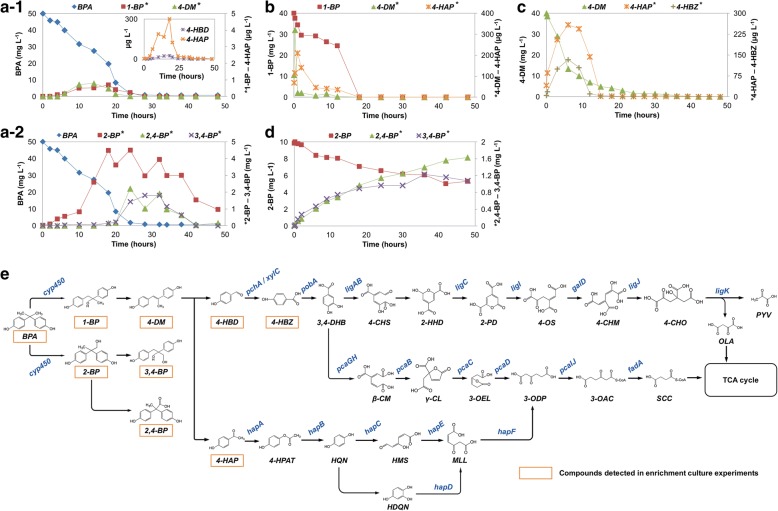
Community-level BPA biodegradation dynamics and pathways. Biodegradation of BPA and intermediates in the enrichment culture amended with (a) 50 mg L^−1^ BPA, (b) 40 mg L^−1^ 1-BP, (c) 40 mg L^−1^ 4-DM, or (d) 10 mg L^−1^ 2-BP. (a-1) 1-BP pathway and (a-2) 2-BP metabolites detected in the enrichment culture amended 50 mg L^−1^ BPA. * The concentrations are indicated using the secondary *Y*-axis. (e) Proposed BPA-mineralization pathways of the enrichment culture. Intermediates marked by orange indicate the degradation products detected by LC-MS/MS from enrichment culture. The details on detection of degradation products are summarized in Additional file 9: Table S1. Genes colored blue were deduced from metagenomic analysis. Abbreviations, BPA, bisphenol A; 1-BP, 1,2-bis(4-hydroxyphenyl)-2-propanol; 2-BP, 2,2-bis(4-hydroxyphenyl)-1-propanol; 4-DM, 4,4′-dihydroxyl-α-methylstilbene; 2,4-BP: 2,2-bis(4-hydroxyphenyl)-propanoate; 3,4-BP, 2,3-bis(4-hydroxyphenyl)-1,2-propanediol; 4-HBD, 4-hydroxybenzaldehyde; 4-HBZ, 4-hydroxybenzoate; 4-HAP, 4-hydroxy-acetophenone; 4-HPAT, 4′-hydroxyphenyl acetate; 4-HPAH, 4-hydroxyphenacyl alcohol; HQN, hydroquinone; 3,4-DHB, 3,4-dihydroxybenzoate; 4-CHS, 4-carboxy-2-hydroxymuconate semialdehyde; 2-HHD, 2-hydroxy-2-hydropyrone-4,6-dicarboxylate; 2-PD, 2-pyrone-4,6-dicarboxylate; 4-OS, 4-oxalome-saconate; 4-CHM, 4-carboxy-2-hydroxy-cis,cis-muconate; 4-CHO, 4-carboxy-4-hydroxy-2-oxoadipate; PYV, pyruvate; OLA, oxaloacetate; HMS, 4-hydroxymuconic semialdehyde; MLL, maleylacetate; β-CM, β-carboxy-muconate; γ-CL, gamma-carboxymucono-lactone; 3-OEL, 3-oxoadipate-enol-lactone; 3-ODP, 3-oxoadipate; 3-OAC, 3-oxoadipyl-CoA; SCC, succinyl-CoA. Gene names are summarized in Additional file 10: Table S6

To characterize the BPA-mineralizing pathway in the enrichment, we monitored the dynamics of BPA and its known degradation intermediates over 48 h in four batch experiments amended with either BPA or one of its previously reported degradation intermediates, i.e., 1-BP, 2-BP, and 4-DM (Fig. 2). These degradation profiles indicate that the community transformed BPA by two divergent pathways via either 1-BP or 2-BP as the respective major intermediate. Further transformation of 1-BP generated 4-DM that was then transformed to either 4-HDB or 4-HAP. 2-BP was further transformed to 2,4-BP and 3,4-BP.

In the experiments with BPA amendment, higher concentrations of 2-BP pathway intermediates were detected than 1-BP pathway intermediates, indicating the accumulation of these intermediates (Fig. 2a). Indeed, comparison of culture amended with either 2-BP or 1-BP showed that 1-BP was more readily and rapidly degradable while 2-BP was more recalcitrant to biodegradation (Fig. 2b, d). When the culture was amended with 10 mg L^−1^ 2-BP or 2,4-BP, accumulation of 2,4-BP and 3,4-BP were also observed during the 48-h incubation. In contrast, the intermediates in the culture amended with 1-BP degraded fairly quickly. Although 4-HBD was observed when the enrichment was amended with 50 mg L^−1^ BPA, it was absent when either 1-BP or 4-DM was amended, indicating the readily degradable nature of these compounds (Fig. 2b, c).

Since the LC-MS/MS analysis did not detect any downstream metabolites potentially involved in the conversion of 4-HBD/4-HAP/2,4-BP/3,4-BP to the intermediates in TCA cycle, we sought to identify the lower pathway of conversion of 4-HBD/4-HAP/2,4-BP/3,4-BP using functional annotation of assembled open reading frames (ORFs) in metagenomics analysis (details described in the next section). These analyses indicate that the BPA-degrading community possessed the genes encoding the transformation of 1-BP pathway downstream intermediates, 4-HBD and 4-HAP (Fig. 2e). 4-HBD could be further transformed to either oxoacetate/pyruvate or succinyl-CoA via 3,4-dihydroxybenzoate (3,4-DHB). 4-HAP could be further transformed to succinyl-CoA via 3-oxoadipate (3-ODP). Currently, it is unclear how 2,4-BP and 3,4-BP are transformed to TCA cycle intermediates. These integrated data of metabolites and metagenomics indicate that the enrichment culture mineralized BPA mainly through the 1-BP pathway via either 4-HDB or 4-HAP to the TCA cycle.

### Integration of metagenomic and metatranscriptomic data to investigate the roles of individual microbial populations in BPA mineralization

To profile the microbial metabolic capability and activity, a total of ~ 36 million metagenomic raw reads (~ 8.8 Gbp), obtained from two different metagenomic libraries, and ~ 256 million metatranscriptomic raw reads (~ 56.4 Gbp), from eight different metatranscriptomic libraries, in addition to ~ 10,000 raw reads of 16S rRNA gene sequences were obtained from biomass samples collected at two different time points from the enrichment culture using 50 mg L^−1^ BPA as the sole substrate (detail of samples and sampling please refer to method sections).

**Fig. 3:**
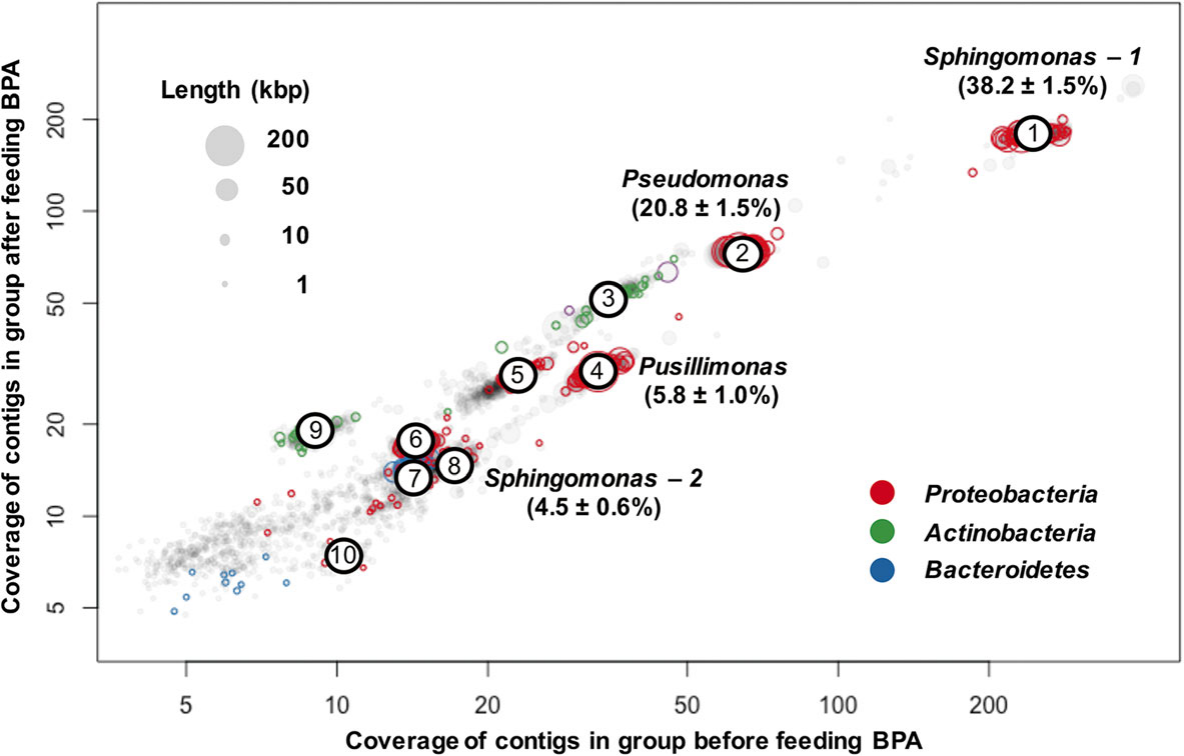
Genomes of dominant species recovered from binning analysis using bi-dimensional coverage plots on metagenomic datasets. Percentage suggests the relative abundance of 16S rRNA gene of the recovered genome in the community

To determine the identities and functions of microbial populations in the enrichment, assembled contigs from metagenomics sequencing were binned using bi-dimensional coverage plots. This analysis identified ten bacterial genomes. While the completeness of seven genomes was higher than 96%, that of the remaining three genomes was below 60% (Fig. 3 and Additional file 1: Table S1). The functions of 62.3 to 92.5% of predicted ORFs of the recovered genomes were identified using BLASTp against NCBI-non-redundant protein sequences, KEGG, and Brenda databases (Additional file 2 and Additional file 3: Table S2). Taxonomic annotation using genome taxonomy database (GTDB) [33] indicated that the genomes belong to eight genera (Additional file 1: Table S1). Two genomes of the genus *Sphingomonas* were found to possess all known genes necessary for degrading BPA. Four genomes from the genera, *Pseudomonas*, *Leucobacter*, *Pusillimonas*, and *Pandoraea*, respectively, contained genes only encoding either full or partial intermediate-degrading pathways (Additional file 1: Table S1).

The 16S rRNA gene 454-pyrosequencing analysis revealed that four bacteria, including two *Sphingomonas* spp*.*, Sph-1 and Sph-2, a *Pseudomonas* sp., and a *Pusillimonas* sp*.*, were the predominantly populations after BPA amendment, accounting for 38.2 ± 1.5%, 4.5 ± 0.6%, 20.8 ± 1.5%, and 5.8 ± 1.0% of relative abundance of community members, respectively (Fig. 3). No obvious abundance shifting of the four strains was observed during the degradation process. Our metatranscriptomic analyses therefore focused on these four strains because of their potential involvement in BPA biodegradation and high abundance as well as high genome completeness and low genetic contamination (≤ 1%)*.* We selected four BPA biodegradation stages: phase I was 24 h after the enrichment culture inoculated into basal medium without BPA; phases II and III were 2 h and 14 h, respectively, after BPA amendment; and phase IV was 24 h after BPA was provided (Fig. 2a).

**Fig. 4:**
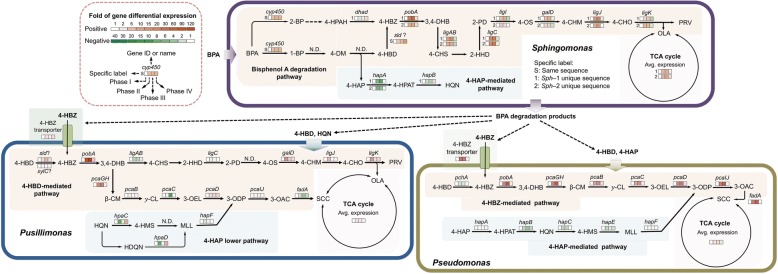
Differential expression of genes involved in the BPA-mineralization process and the pattern of substrate cross-feeding between BPA-degrading *Sphingomonas* spp*.* and BPA non-degraders *Pseudomonas* sp. and *Pusillimonas* sp. Specific label “S,” “1,” and “2” indicates Sph-1 and Sph-2 share the same sequence between each other, Sph-1 unique sequence and Sph-2 unique sequence, respectively

Metagenomic analysis identified 3707 and 6077 ORFs in Sph-1 and Sph-2, respectively (Additional file 2). Both Sph-1 and Sph-2 contained genes encoding enzymes predicted to convert BPA to 1-BP and 2-BP. Nine putative genes were predicted to encode cytochrome P450 enzymes (CYP) in Sph-1 and Sph-2. One of the proposed CYP gene that was present in both genomes shares 99% similarity in nucleotide sequence (across 98% of its full-length 1290 bps) with a CYP gene that was previously characterized as the principal BPA-degrading enzyme of *Sphingomonas* sp. AO1 [34]. The same scaffold where the CYP gene was found also contained the other component of the BPA-degrading enzyme system, a ferredoxin gene which shares 100% similarity with the ferredoxin from the strain AO1 [34]. Although the degradation reactions have been reported after the initial transformation of BPA to 1-BP or 2-BP, the enzymes involved in these steps are still unknown. The two *Sphingomonas* genomes also carried the genes encoding the conversion of 4-HBZ to oxaloacetate/pyruvate and 4-HAP to 4-HPAT (Fig. 4). Specific genes encoding the conversion of 4-HPAT to hydroquinone (HQN), and 4-hydroxyphenacyl alcohol (4-HPAH) to 4-HBZ, were only present in Sph-1. The gene encoding 4-HBD conversion seemed to be absent from either *Sphingomonas* genome. Instead, a gene was found in both *Sphingomonas* genomes to transform salicyladehyde, a 4-HBD isomer, indicating novel pathways might be involved in the conversion of 4-DM to 4-HBZ.

To our knowledge, the two *Sphingomonas* genomes recovered herein were the first two reported draft genomes of BPA-degrading *Sphingomonas* spp. To achieve a better understanding of their phylogenetic relationship with other *Sphingomonas* spp*.*, nearly full-length 16S rRNA gene sequences were obtained from 16S rRNA gene cloning. Phylogenetic analysis indicates both Sph-1 and Sph-2 were distantly related to each other and to previously reported BPA-degrading *Sphingomonas* spp*.* (Additional file 2 and Additional file 3: Figure S1).

Metatranscriptomics analysis of Sph-1 and Sph-2 indicated that there were two major response groups of the BPA pathway genes that we identified as A (arched)-shaped expression patterns (up-regulated in phases II and III; flat or downregulated in phase IV) and U-shaped expression patterns (downregulated in phases II and III; flat or upregulated in phase IV). The genes encoding the CYP and the ferredoxin exhibited consistent A-shaped patterns, indicating their important roles in BPA biodegradation (Fig. 4). Similarly, the expression of most genes in the 4-HBZ to pyruvate/oxaloacetates pathways (*pobA*, *ligAB*, *ligC*, *galD*, *ligJ*, and *ligK*) and the TCA cycle genes were also A-shaped (Fig. 4). In contrast, *hapA* and *hapB*, involved in the conversion of 4-HAP to HQN were U-shaped as was *dhad*, indicating the inactivity of these genes in our experiments. Interestingly, although the genes involved in 2-BP degradation are unknown, a few oxidoreductases were upregulated only in the final phase where 2-BP pathway intermediates were dominant, suggesting their potential correlation to 2-BP degradation (Additional file 2 and Additional file 4: Figure S2). These analyses suggest that Sph-1 and Sph-2 were the major BPA-degrading populations in the community.

Metagenomic analysis indicated that the *Pseudomonas* and *Pusillimonas* in the enrichment contained 5672 and 4050 ORFs, respectively (Additional file 2). The *Pseudomonas* sp. lacked genes responsible for initial BPA degradation to either 1-BP or 2-BP but possessed a complete pathway that converts 4-HBD to succinyl-CoA and the gene encoding the 4-HBZ transporter across the membrane (Fig. 4). Similarly, the *Pusillimonas* sp. possessed two almost complete pathways for converting 4-HBZ to either oxaloacetate/pyruvate or succinyl-CoA and the 4-HBZ transport gene (Fig. 4). Also, the *Pseudomonas* genome contained a complete pathway for the transformation of 4-HAP to 3-ODP. The *Pusillimonas* genome encoded a lower 4-HAP pathway converting HQN to 3-ODP, but the genes responsible for transformation of 4-HAP to HQN were missing.

*Pseudomonas* sp. exhibited A-shaped patterns for those genes involved in 4-HBZ transporter, conversion of 4-HBZ to succinyl-CoA, and the TCA cycle. The genes for converting 4-HBD to 4-HBZ (*pchA*) and 4-HAP to 3-ODP (*hapA*, *hapB*, *hapC*, *hapE*, *hapF*) were U-shaped, suggesting they might not participate in BPA biodegradation under the tested conditions (Fig. 4). Unlike *Pseudomonas* sp., *Pusillimonas* sp. exhibited inconsistent expression patterns for genes encoding either pathways for the conversion of 4-HBZ downstream intermediates, suggesting that it might use a hybrid pathway to mineralize 4-HBZ. The lower 4-HAP-degrading pathway (from HQN to succinyl-CoA) also exhibited U-shaped regulation. The upregulation of genes in this pathway in phase IV indicates their functions at the late stage of BPA biodegradation (Fig. 4).

The integration of 16S-sequencing, metagenomic, and metatranscriptomic analyses revealed an interesting metabolic interdependence between *Sphingomonas* spp. and *Pseudomonas* sp. or *Pusillimonas* sp. (Fig. 4). This interaction model suggests that two *Sphingomonas* species were the key BPA-degraders, converting BPA to 4-HBZ and other intermediates that likely supported the growth of non-degrading microbial populations*.* (Fig. 4).

### Confirmation of microbial interactions in BPA biodegradation using bacterial isolates and consortium from the BPA-degrading community

To determine if the hypothesized substrate cross-feeding played a role in the BPA-degrading efficiency, we designed isolation strategies in order to capture both the BPA-degrading and lower pathway metabolite-utilizing bacteria by using either BPA-containing or non-selective medium. This approach isolated a *Sphingomonas* sp. and a *Pseudomonas* sp. from the BPA-containing and non-selective medium, respectively. Genomic analysis of the draft genome of these isolates showed the average nucleotide identities were 100 ± 0.48% and 100 ± 0.04% similar to the binned genomes of Sph-2 and *Pseudomonas* sp. respectively.

**Fig. 5:**
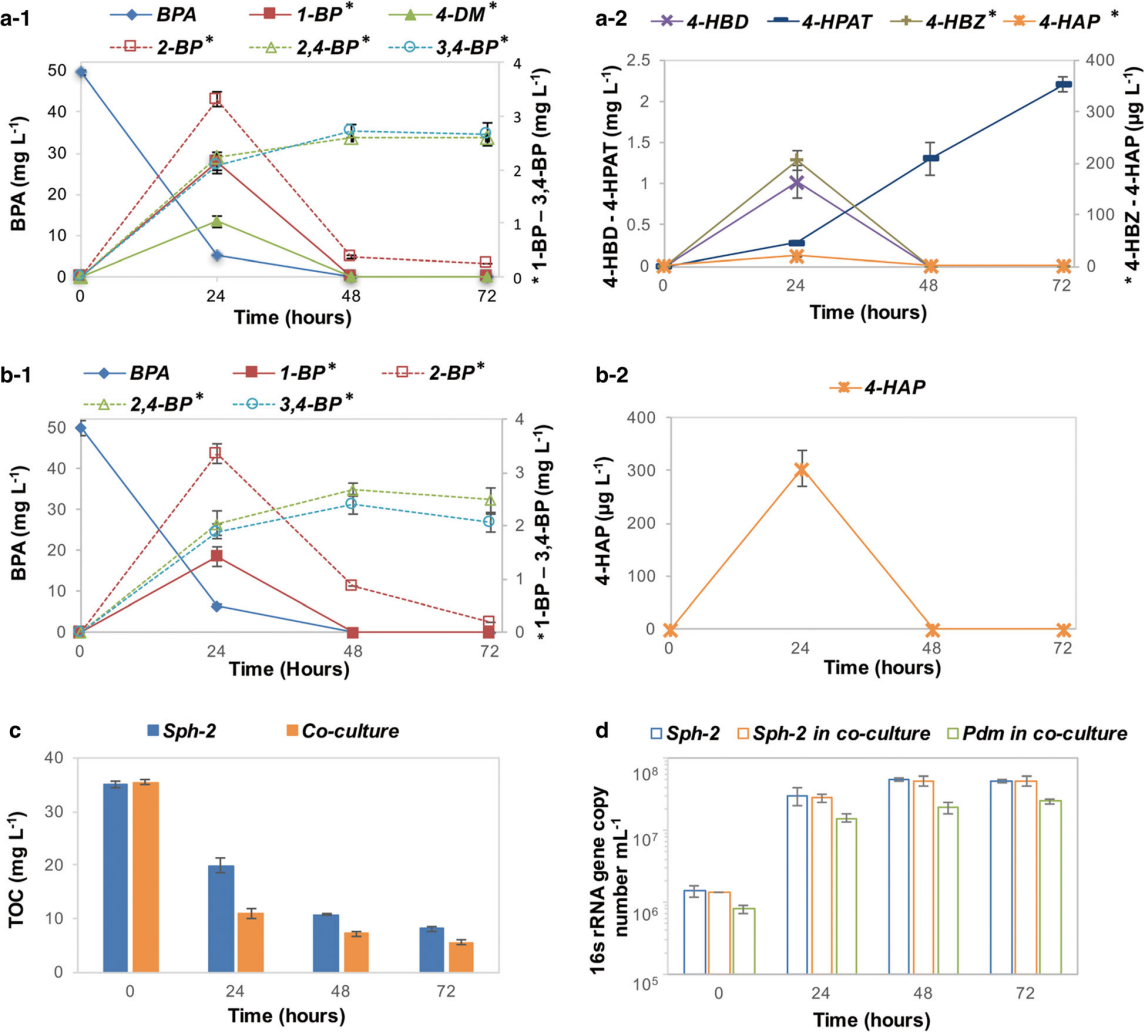
Biodegradation behavior and growth of Sph-2 in axenic culture and in co-culture with *Pseudomonas* sp. Biodegradation of BPA by Sph-2 axenic culture (a) or Sph-2*/Pseudomonas* sp. co-culture (**b**); total organic carbon detected in Sph-2 axenic culture and co-culture with *Pseudomonas* sp. (**c**); cell growth detected in Sph-2 axenic culture and co-culture with *Pseudomonas* sp. (**d**). Error bars indicate the standard deviation of biological triplicates

In batch axenic culture, Sph-2 quickly degraded BPA, 1-BP, 4-DM, 4-HBD, and 4-HBZ, but slowly degraded 2-BP and 4-HAP. Sph-2 was inefficient at degrading 2,4-BP, 3,4-BP, and 4-HPAT that were accumulative during the incubation (Fig. 5a and Additional file 5: Figure S3a, b). These results are in agreement with the downregulation of the genes for conversion of 4-HAP and 4-HPAT, and upregulation of genes involved in 4-HBD and 4-HBZ degradation (Fig. 4). Consistent with our prediction from the integrated meta-omics analysis, *Pseudomonas* sp. was efficient at degrading 4-HBZ, but not BPA, 1-BP, 4-DM, and 2-BP. *Pseudomonas* sp. also demonstrated the abilities to degrade 4-HBD, 4-HAP, and 4-HPAT with higher efficiencies to degrade 4-HBD and 4-HBZ than 4-HAP and 4-HPAT (Additional file 5: Figure S3c), but lacking capacity to degrade BPA, 1-BP, 2-BP and 4-DM, which is consistent with metagenomics prediction. The downregulation of genes involved in the transformation of 4-HAP and 4-HPAT (*hapA and hapB*) observed in the community probably reflected that they were less favorable for *Pseudomonas* sp.

The co-culture of Sph-2 and *Pseudomonas* sp. demonstrated faster and more complete BPA mineralization than the Sph-2 axenic culture, even though comparable BPA degradation rates were observed in both sets of cultures (Fig. 5a–c). For example, after 24-h incubation, about 69 ± 0.5% of TOC was removed in the co-culture, whereas only about 40 ± 0.6% disappeared in the axenic culture. At the end of the incubation (72 h), about 84 ± 0.4% and 77 ± 0.4% of TOC were found in the co-culture and axenic culture, respectively. The higher TOC removal efficiency, especially in the first 24 h, observed in the co-culture was related to the disappearance of intermediates such as 1-BP, 4-DM, 4-HBD, 4-HBZ, 4-HAP, and 4-HPAT. In fact, 4-HBD, 4-HBZ, and 4-HPAT were not detected in the co-culture, indicating their fast degradation by *Pseudomonas* sp. (Fig. 5b). Although *Pseudomonas* sp. was incapable of degrading 4-DM, 4-DM was not detected in the co-culture. The disappearance of 4-DM was probably caused by a faster consumption by Sph-2 as a result of the fast removal of 4-DM downstream metabolites by *Pseudomonas* sp. In agreement with the fast utilization of BPA and intermediates, both cell numbers of Sph-2 and *Pseudomonas* sp. increased significantly over 24 h. Similar Sph-2 cell numbers ((1.4 ± 0.4) × 10^6^ cell mL^−1^) were inoculated in both sets of cultures while 3.2 ± 0.3 × 10^5^*Pseudomonas* were inoculated to the co-culture to mimic the relative abundance observed in the enrichment (Fig. 3a). Sph-2 increased to the similar amount (4.8–5.0 ± 0.8 × 10^7^ cell mL^−1^) in both of the co-culture and the Sph-2 axenic culture, while the *Pseudomonas* sp. increased to 3.3 ± 0.7 × 10^7^ cell mL^−1^ after 72-h incubation (Fig. 5d). These results indicate that even though *Pseudomonas* sp. consumed BPA degradation products from Sph-2, it did not affect the growth of Sph-2.

## Discussion

**Fig. 6:**
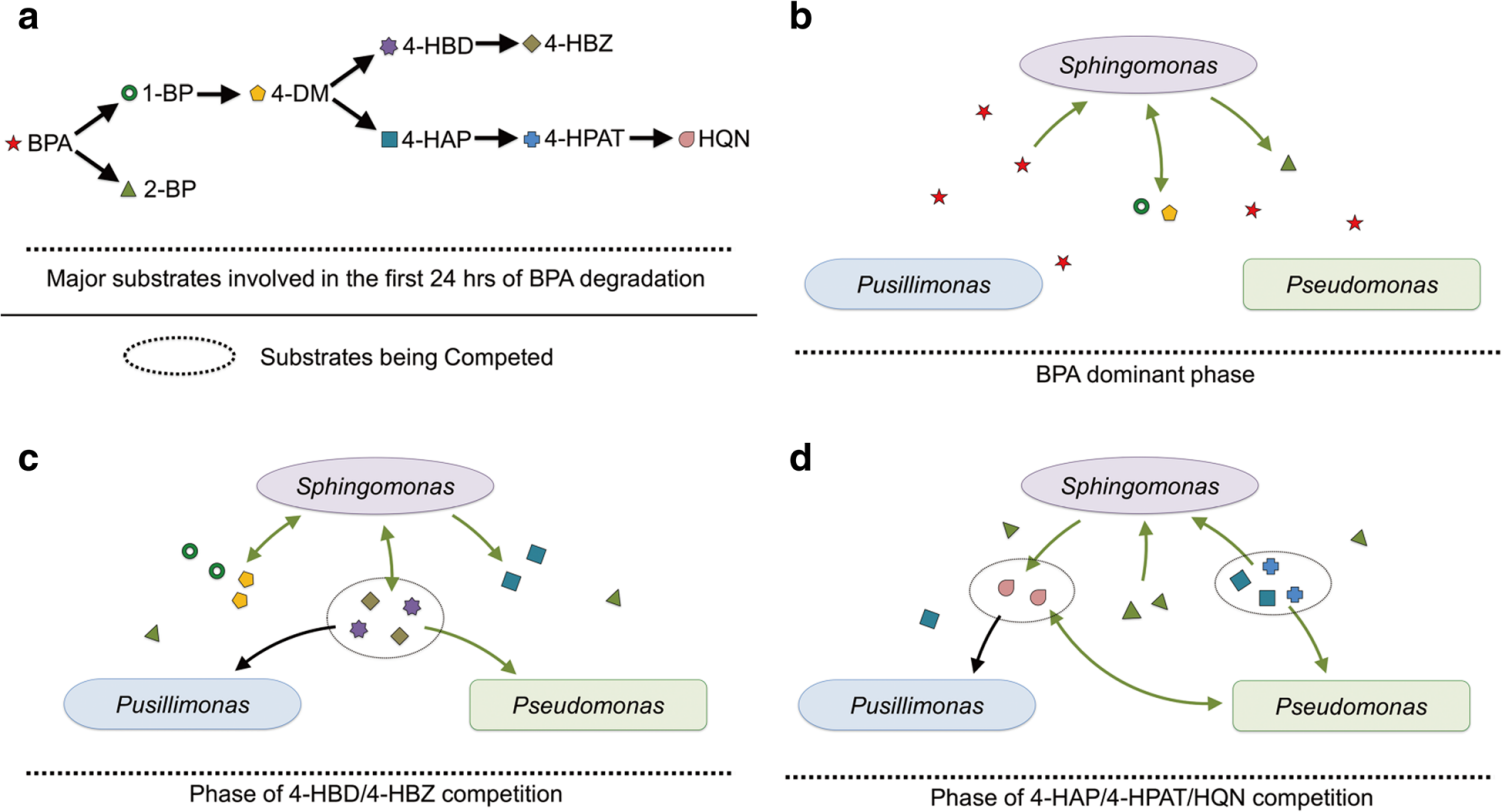
Proposed substrate cross-feeding between BPA-degrading *Sphingomonas* sp. and non-degrading *Pseudomonas* sp. or *Pusillimonas* sp. A simplified pathway presentation of major substrates found in the bulk community environment (**a**); *Sphingomonas* sp. in the community transformed BPA to 1-BP, 4-DM, and 2-BP during the initial stage of biodegradation (**b**); then further transformed 1-BP and 4-DM to either 4-HBD or 4-HAP; 4-HBD was quickly converted to 4-HBZ; both of 4-HBD and 4-HBZ were used by *Pseudomonas* sp. and *Pusillimonas* sp. (**c**); and *Sphingomonas* started consuming 2-BP after 4-HBD/4-HBZ depletion, while *Pseudomonas* coverts 4-HAP to 4-HPAT and then HQN (**d**); HQN was degraded by the *Pusillimonas*. Lines with green arrow suggests the interaction was confirmed by experiment by using isolates

The enhancement of BPA biodegradation of *Sphingomonas* sp. by a non-degrader *Pseudomonas* sp. was previously observed but the underlying mechanism supporting the enhancement was unknown [32]. The analyses of this study showed that though *Sphingomonas* spp*.* could completely degrade BPA, this degradation was inefficient since intermediates accumulated (Fig. 5a). During the initial phase of BPA degradation, *Sphingomonas* spp*.* converted BPA to 1-BP and 2-BP and then preferentially degraded 1-BP to 4-DM that was further converted to 4-HBD and 4-HAP (Fig. 6b). Interestingly, though *Sphingomonas* spp*.* clearly retained sufficient 4-HBD and 4-HBZ to sustain their growth, they degraded these downstream intermediates of 4-DM less efficiently than the other community members. On the other hand, non-degraders, e.g., the *Pseudomonas* sp., could utilize the intermediates more quickly, therefore facilitated the overall BPA mineralization in the community or in the co-culture consortium (Fig. 6c). Although 4-HAP was probably not a preferable substrate for *Pseudomonas* in comparison to 4-HBD/4-HBZ, *Pseudomonas* sp. also contributed significant degradation of 4-HAP after 4-HBD/4-HBZ depletion in the microbial community. *Pusillimonas* sp. also possessed 4-HBZ transporters similar as *Pseudomonas* sp.; however, it might be less competitive for this substrate as evidenced by the inconsistent expression levels of genes involved in the 4-HBD/4-HBZ-degrading pathway and lower population abundance in the community. *Pusillimonas* sp. may have participated in degradation of the downstream intermediate of 4-HAP, e.g., HQN at the later stage of BPA biodegradation as shown by the upregulation of gene expression (Figs. 4 and 6d).

An associated metabolism based on the cross-feeding with metabolites of the BPA degradation pathway was an important microbial interaction that enabled the microbial community to extract the maximum carbon and energy from the given substrate, BPA. Such cooperative cross-feeding was also observed in other microbial communities degrading aromatic compounds [35–38]. Collectively, these studies indicate that though the key degrading bacteria are important for the biodegradation of targeted xenobiotics, they are not solely responsible for the effective performance of a microbial community. The populations involved in the degradation of intermediates are also important. In the environmental bioprocesses, such as wastewater treatment and bioremediation, bioaugmentation, a practice of adding microorganisms that are capable of degrading specific compounds to existing biomass, is sometimes necessary when biodegradation population is absent at the contaminated site [39]. An important implication of understanding the cooperative metabolism is that bioaugmentation of a community or consortium containing the cooperative metabolic groups might be more advantageous than the biodegrading bacterium alone.

Additionally, our study demonstrated that metagenomics data were inadequate to resolve a complete biodegradation pathway in BPA-biodegrading microbial communities due to the limited understanding of biodegradation enzymes. Currently, only CYP involved in conversion of BPA to 1-BP or 2-BP was identified and characterized [34]. The enzymes involved in the rest of the conversion of 1-BP to 4-HBZ are still unknown. On the other hand, metabolite analysis by LC-MS/MS alone cannot show the complete BPA mineralization either. Many transient metabolites in the lower pathway were unable to be detected using the LC-MS/MS analysis in this study. The combination of metagenomic annotation with metabolites analysis therefore represents an efficient way to obtain a comprehensive understanding of metabolic capability, which paves the way for examining the relativeness of metabolic capability of each individual population to the community as a whole.

## Conclusion

In summary, we demonstrated that the three types of integrated meta-omics analyses could effectively reveal the metabolic capability at both community-wide level and individual bacterial level. The further correlation between these two levels reveals the hidden connection between apparent overall community performance and the contribution of individual community members and their synergy in a BPA-degrading microbial community. Furthermore, we have demonstrated that using an integrated multi-omics technique, in conjunction with a culture-based confirmation approach, can effectively reveal the microbial interactions that affect the performance outcome. We foresee that this approach would contribute to the future application and operation of environmental bioprocesses on a knowledge-based control.

## Methods

### Chemicals

Chemical standards, including BPA, 4-hydroxylbenzaldehyde (4-HDB), 4-hydroxybenzoate (4-HBZ), 4-hydroxylacetophenone (4-HAP), 4′-hydroxyphenyl acetate (4-HPAT), and hydroquinone (HQN), were purchased from Sigma-Aldrich (CA, USA). Other standard compounds, including 1,2-bis(4-hydroxyphenyl)-2-propanol (1-BP) and 2,2-bis(4-hydroxyphenyl)-1-propanol (2-BP), 2,2-bis(4-hydroxyphenyl)-propanoate (2,4-BP), and 4,4′-dihydroxyl-α-methylstilbene (4-DM), were synthesized by Richest Company (Shanghai, China). The purity of all standards was higher than 98%. Additional file 6: Table S2 summarizes a list of chemicals, their names, structures, and abbreviations used in this study.

### Culture medium, enrichment, isolation, and batch biodegradation experiments

BPA medium was prepared with basal salt medium as described previously [30]. The BPA-degrading microbial community used in this study was enriched from activated sludge obtained from a wastewater treatment plant in Hong Kong, China. Briefly, 10% of activated sludge was inoculated into a 250-ml flask containing 100 ml BPA medium that was amended with 20 mg L^−1^ BPA. After each dose of BPA was used, the enrichment culture (5% *v*/*v*) was sub-cultured in new medium with 20 mg L^−1^ BPA every 5 days at room temperature with shaking at 150 rpm for 2 months. The culture was then transferred to the medium containing 50 mg L^−1^ BPA and sub-cultured for another 3 months. To investigate the biodegradation behaviors of the enrichment culture, BPA or the biodegradation intermediates were amended as the sole carbon and energy source for the enrichment culture. To isolate BPA-degrading and non-degrading bacteria from the enrichment culture, either BPA medium or R2A medium was used.

Batch experiments amended with BPA or its degradation intermediates (1-BP, 2-BP, 4-DM, 2,4-BP, 4-HBD, 4-HBZ, 4-HAP, or 4-HPAT) were constructed to examine the degradation capability of the enrichment culture, isolates, and co-culture of isolates. Cell number in the pure or co-cultures was determined by quantitative real-time PCR (qPCR) with primers specific to the 16S rRNA genes of two isolates using a StepOnePlus real-time PCR system (Life Technologies, NY, USA) as described previously (Additional file 7: Table S3) [4]. Since only one copy of 16S rRNA gene was found on the two isolates, cell number was equal to the 16S rRNA gene copies measured by qPCR.

### BPA biodegradation product analysis using LC-MS-MS

To understand the BPA biodegradation capabilities of the enrichment, pure culture, and co-cultures, targeted metabolite analysis was performed on the batch experiments amended with either BPA (50 mg L^−1^) or its known transformation products, including 1-BP (50 mg L^−1^), 2-BP (10 mg L^−1^), 4-DM (50 mg L^−1^), and 2,4-BP (10 mg L^−1^) (Additional file 8: Table S4). Batch samples were filtrated with polyvinylidene fluoride membrane (0.22 μm) and diluted three to five folds to avoid signal suppression caused by matrix effect.

BPA and its transformation intermediates were analyzed with an ultra-performance liquid chromatography-triple quadrupole mass spectrometer (Acquity™_,_ Waters, Manchester, UK). Briefly, samples were injected onto a BEH™ C_18_ column (1.7 μm, 50 mm × 2.1 mm) (Waters, Manchester, UK). The flow rate was 0.3 ml min^−1^, and elution solvents consisted of 5% methanol in water (solvent A) and 5% water in methanol (solvent B). Samples were eluted with a solvent program that was started at 5% solvent B, increased to 95% solvent B in a linear gradient over 3 min, and remained at 95% solvent B for 6 min. The tandem triple quadrupole mass spectrometry was set for multiple reaction monitoring for quantification of BPA and its intermediates in negative electrospray ionization mode with the cone voltage set to 45 V and the collision energy set to 20 eV.

### DNA and RNA sequencing

In the experiments amended with BPA, DNA and RNA sampling time points were divided into four phases. Phase I was the time before PBA was introduced to the enrichment culture. Phases II and III were 2 h and 14 h, respectively, after BPA was provided. Phase IV was 24 h after BPA was provided. Two DNA samples from phases I and III, respectively, were collected and extracted for metagenomics and 16S rRNA gene-tag sequencing. Total eight RNA samples, i.e., duplicate RNA samples from each phase, were extracted for metatranscriptomic sequencing. Collected samples of DNA and RNA were summarized in Additional file 9: Table S5.

DNA extraction was performed on biomass pellet that was collected from 50 mL samples after centrifuge at 13,000*g* for 10 min at 4 °C using FastDNA SPIN Kit for Soil (Q-Biogene, CA, USA) following manufacturer’s instruction. Total RNA extraction was carried out with PowerSoil Total RNA Isolation Kit (MO-BIO Laboratories, Inc., CA, USA) as described previously [16]. Ribosomal RNA was removed by Ribo-Zero™ rRNA removal kits (Epicentre, WI, USA) following the manufacturer’s instruction.

For sequencing both the community metagenomes and the genomes of bacterial isolates, paired-end and fragment libraries of DNA and cDNA were prepared following Illumina manufacturer’s instruction. DNA and cDNA fragment libraries (~ 200 bp) were constructed for metagenomic and metatranscriptomic sequencing using Illumina HiSeq 2000 platform (Illumina, CA, USA). The base-calling pipeline was used to process the raw fluorescence images and call sequences. Length of raw read was trimmed to 100 bp for each read. Raw reads with > 10% unknown nucleotides or with > 20% low-quality nucleotides (quality value < 20) were discarded.

### Metagenomics assembly, binning, taxonomic annotation and functional annotation

The trimmed paired-end reads from the two metagenomes were assembled individually and co-assembled by CLCbio de novo assembly algorithm, using a k-mer of 63 and a minimum contig length of 1 kbp. Reads from two metagenomes were then individually mapped to scaffolds using CLCbio with a minimum similarity of 98% over 100% of the read length. The relative metagenome abundance of each genome bin was calculated as a percentage of metagenome reads mapped to a specific bin in the total metagenome reads.

A bi-dimension binning process was applied to recover the genomes of dominant species from metagenomic datasets and the three assemblies using a R script package [40]. Briefly, coverage of each scaffold was calculated and all scaffolds were further grouped by bi-dimension coverage to recover potential bins. Scaffolds belonging to the certain bin were further clustered by tetranucleotide frequency to remove contamination. Paired-end tracked scaffolds were utilized to retrieve multiple-copy genes. The recovered bins were extracted from the scaffold pool as binned genomes. Filtrated reads from isolations sequencing were also utilized for assembly (CLCbio) individually. CheckM (version 1.0.11) was used to evaluate the genome completeness using marker genes [41]. Genomes of *Sphingonomas* (Sph-1 and Sph-2), *Pseudomonas*, and *Pusillimonas* were selected to carry out metatranscriptomics analysis and further prediction of bacterial interaction because of their potential dominant roles in the mixed community as well as the estimated high completeness and quality of these genomes (> 95%) and low genomic contamination (< 1%).

Scaffolds were submitted to MetaProdigal (version 2.6.3) [42] for open reading frame (ORF) calling. ORFs were further BLASTx against KEGG and NCBI-nr database with an *e* value of 1e^−5^ for functional annotation. Integration and visualization of KEGG blast results were performed by Pathview (version 3.5) [43]. Simultaneously, functional prediction of novel sequences was performed by Pfam (version 31) [44]. Sequences from Brenda database were also being used to replenish the annotation results [45]. Annotation outputs were further manually checked.

Taxonomic annotation was carried out by annotating the recovered bin-genomes to genome taxonomy database using GTDB toolkits (version 1.3) [33]. Scaffolds of recovered genomes were also blast against SILVA SSUref (version 128) database for taxonomic annotation.

### Pathway reconstruction of BPA degradation pathway by metagenomics

The possible BPA degradation pathways in the dominant species in the community, which were characterized by metagenomics, were utilized to predict the UPLC-MS/MS undetected metabolic products. The prediction of possible degradation pathways was based on the known paired relationship between genes, substrates, and products in available databases (e.g., KEGG database). Briefly, once the functional gene involved in a certain physiological process was detected from the community, the possible substrate and product would be predicted accordingly.

### 16S rRNA-tag pyrosequencing

Four DNA samples (two samples from each before and 15 h after BPA (50 mg L^−1^) amendment) were used to perform pyrosequencing of 16S rRNA-tag analysis. V3 and V4 regions of 16S rRNA gene sequences were amplified from the DNA extracts and cDNA samples using the primer set of forward (5′-ACTCCTACGGGAGGCAGCAG-3′) at the 5′-end of the V3 region and a cocktail of four equally mixed reverse primers, which were R1 (5′-TACCRGGGTHTCTAATCC-3′), R2 (5′-TACCAGAGTATCTAATTC-3′), R3 (5′-CTACDSRGGTMTCTAATC-3′), and R4 (5′-TACNVGGGTATCTAATCC-3′), at the 3′-end of the V4 region. Amplicons were purified with a quick-spin Kit (iNtRON, Seoul, Korea), and concentrations were measured by NanoDrop nv-Vis spectrophotometer (Thermo, USA). Triplicate independent PCR products were prepared for each amplicon library to reduce the impact of potential early-round PCR errors. Amplicons from different samples were then mixed to achieve equal mass concentrations in the final mixture, which was sent out for pyrosequencing on the Roche 454 FLX Titanium platform (Life Technologies, NY, USA).

### Metatranscriptomics analyses and analysis of active BPA-mineralization pathways

Duplicate RNA samples were collected at four phases for metatranscriptomics analyses (Additional file 9: Table S5). After quality control analysis, RNA sequences were aligned against contigs or predicted ORFs for differential expression analysis of rRNA or mRNA gene using BOWTIE2 [46]. Gene expression level was represented by RPKM (reads per kilobase per million mapped reads) value as described previously [47]. For comparison of gene expression level of genes from the same genome, RPKM value of gene were calculated by mapped read of the targeting gene against number of total mapped reads of the same genome.

To exclude the bias of comparison of gene expression level caused by cellular growth, we normalized the number of mapped reads of a certain ORF against mapped reads of total ORFs from a certain genome for RPKM value calculation [48]. For instance, if gene *a* is present in genome A, the RPKM value will be calculated by the following equation:

$$ \mathrm{RPKM}=10\hat{\mkern6mu} 6\times a/\left(A\times l\right) $$

where *a* is total number of mapped reads of a gene, *A* is the total mapped reads of A genome, and *l* is the length of the gene. The normalized RPKM value helps to better compare gene expression level among genes from the same genome, rather than from the whole community.

## Additional files

Additional file 1: **Table S1.** Genomic information of dominant species that were recovered from binning. (XLSX 33 kb)

Additional file 2: Supplementary methods. (DOCX 38 kb)

Additional file 3: **Figure S1.** 16S rRNA-based phylogenetic tree of Sph-1 and Sph-2 suggests the two genomes from two different *Sphinomonas* species. Genomes with yellow background color suggest previously reported BPA-degrading *Sphingomonas*. (PDF 2537 kb)

Additional file 4: **Figure S2.** Heat map matrix of gene expression of oxidases (a) with identical sequences in Sph-1 and Sph-2; (b) unique to Sph-1; (c) unique to Sph-2; (d) in *Pseudomonas*; (e) in *Pusillimonas*. (f) Percentage of mapped reads in total reads of the ORFs predicted from four species and total predicted ORFs in different phases. Abbreviation indicates the enzymes coding genes possibly involved in BPA degradation process. *p450*, cyp450 encoding sequence (* suggest the cyp450 sequence involved in initial reaction of BPA degradation); *dhbzA*, protocatechuate 4,5-dioxygenase; *dhbzB*, protocatechuate 3,4-dioxygenase; *oor*, 2-oxoacid:ferredoxin oxidoreductase; *hbmo*, 4-hydroxybenzoate 3-monooxygenase; *mhpB*, 3-(2,3-dihydroxyphenyl)propionate dioxygenase; *dmpB*, catechol 2,3-dioxygenase; *hppD*, 4-hydroxyphenylpyruvate dioxygenase; *dhad*, 2,4′-dihydroxyacetophenone dioxygenase; *qodI*, quercetin 2,3-dioxygenase; *co*, carotenoid oxidase; *hqd*, hydroxyquinol 1,2-dioxygenase; *hapD*, hydroquinone dioxygenase; *ben*, benzene 1,2-dioxygenase; *ccdo*, catechol 1,2-dioxygenase; *hapA*, 4-hydroxyacetophenone monooxygenase; *bphd*, biphenyl 2,3-dioxygenasel; *tauD*, taurine dioxygenase; *mqo*, malate:quinone reductase (EC 1.1.5.4); *nuor*, NADH:ubiquinone oxidoreductase; *phyH*, phytanoyl-CoA dioxygenase; *hgd*, homogentisate 1,2-dioxygenase (*EC 11.13.11.5*); *pdo,* phthalate dioxygenase (*EC 1.14.12.7*); *phyH*, phytanoyl-CoA dioxygenase (EC 1.14.11.18); *hdq*, hydroxyquinol 1,2-dioxygenase. (PDF 7039 kb)

Additional file 5: **Figure S3.** (a) Biodegradation of 1-BP, 4-DM and 2-BP in *Sphingomonas sp.* axenic culture. Biodegradation of 4-HBD, 4-HBZ, 4-HAP and 4-HPAT in *Sphingomonas sp.* axenic culture (b); and (c) *Pseudomonas sp.* axenic culture. “Sph-2” and “Pdm” indicates *Sphingomonas sp.* and *Pseudomonas sp.*, respectively. Error bars indicate the standard deviation of biological triplicates. (PDF 1706 kb)

Additional file 6: **Table S2.** Summary of standard bisphenol A and bisphenol A degradation products. (XLSX 425 kb)

Additional file 7: **Table S3.** Primer sets used in qPCR analysis. (XLSX 28 kb)

Additional file 8: **Table S4**. Determination of major degradation products of BPA in partial batch experiments that fed with either BPA or its metabolic products. (XLSX 31 kb)

Additional file 9: **Table S5.** DNA and RNA samples collected from 50 mg L^−1^ BPA batch. (XLSX 32 kb)

Additional file 10: **Table S6.** Summary of genes encoding enzymes involved in conversation of bisphenol A degradation products Supplementary Table S6. Summary of genes encoding enzymes involved in conversation of bisphenol A degradation products. (XLSX 34 kb)

## Abbreviations

1-BP: 1,2-Bis(4-hydroxyphenyl)-2-propanol
2,4-BP: 2,2-Bis(4-hydroxyphenyl)-propanoate
2-BP: 2,2-Bis(4-hydroxyphenyl)-1-propanol
2-HHD: 2-Hydroxy-2-hydropyrone-4,6-dicarboxylate
2-PD: 2-Pyrone-4,6-dicarboxylate
3,4-BP: 2,3-Bis(4-hydroxyphenyl)-1,2-propanediol
3,4-DHB: 3,4-Dihydroxybenzoate
3-OAC: 3-Oxoadipyl-CoA
3-ODP: 3-Oxoadipate
3-OEL: 3-Oxoadipate-enol-lactone
4-CHM: 4-Carboxy-2-hydroxy-cis,cis-muconate
4-CHO: 4-Carboxy-4-hydroxy-2-oxoadipate
4-CHS: 4-Carboxy-2-hydroxymuconate semialdehyde
4-DM: 4,4′-Dihydroxyl-α-methylstilbene
4-HAP: 4-Hydroxy-acetophenone
4-HBD: 4-Hydroxybenzaldehyde
4-HBZ: 4-Hydroxybenzoate
4-HPAH: 4-hydroxyphenacyl alcohol
4-HPAT: 4′-Hydroxyphenyl acetate
4-OS: 4-Oxalome-saconate
BPA: Bisphenol A
*fadA*: 3-Oxoadipyl-CoA thiolase
*galD*: 4-Oxalomesaconate tautomerase
*hapA*: 4-Hydroxyacetophenone monooxygenase
*hapB*: 4-Hydroxyacetophenone hydrolase
*hapC*: Hydroquinone dioxygenase
*hapD*: Hydroxyquinol 1,2-dioxygenase
*hapE*: 4-Hydroxymuconic semialdehyde dehydrogenase
*hapF*: Maleylacetate reductase
HMS: 4-Hydroxymuconic semialdehyde
HQN: Hydroquinone
*ligAB*: 4-Hydroxybenzoate 3-monooxygenase
*ligC*: 2-hydroxy-4-carboxymuconate semialdehyde hemiacetal dehydrogenase
*ligI*: 2-Pyrone-4,6-dicarboxylate lactonase
*ligJ*: 4-Oxalomesaconate hydratase
*ligK*: 4-Hydroxy-4-methyl-2-oxoglutarate aldolase
MLL: Maleylacetate
OLA: Oxaloacetate
*pcaB*: γ-Carboxymucono-lactone hydrolase
*pcaC*: 4-Carboxymuconolactone decarboxylase
*pcaD*: 3-Oxoadipate-enol-lactonase
*pcaGH*: Protoascatechuate 3,4-dioxygenase
*pcaIJ*: 3-Oxoadipate CoA-transferase
*pchA*: 4-Hydroxybenzaldehyde dehydrogenase
PYV: Pyruvate
SCC: Succinyl-CoA
*sld*: Salicylaldehyde dehydrogenase
*xylC*: Benzaldehyde dehydrogenase
β-CM: β-Carboxy-muconate
γ-CL: Gamma-carboxymucono-lactone
