# Supplementary material for: An integrated meta-omics approach reveals substrates involved in synergistic interactions in a bisphenol A (BPA)-degrading microbial community
Source: Microbiome. 2019 Feb 6;7:16. doi: 10.1186/s40168-019-0634-5 (PMC6366072; doi:10.1186/s40168-019-0634-5)
Supplement: Supplementary file 2 — Supplementary methods. (DOCX 38 kb) [file 40168_2019_634_MOESM2_ESM.docx]

**Supplementary Methods**

**16S rRNA gene cloning.** For calculating the relative abundance of targeted community members, we correlated 16S rRNA gene sequences obtained from 16S rRNA-tag pyrosequencing to those recovered from the dominant binned genomes (Fig. 1). 16S rRNA gene cloning was performed for the two *Sphingomonas* *spp.*to obtain full-length of 16S rRNA genes to achieve accurate phylogenetic analysis. Totally, 30 colonies were selected and sequenced using ABI 3730 automated sequencer (Life Technologies, NY, USA).

**Comparison of the genomes recovered from binning and isolated *Sphingomonas sp.* and *Pseudomonas sp.*.** The genomes of the isolated *Sphingomonas sp.* and *Pseudomonas sp.* were compared with genomes recovered from metagenomic binning. The average nucleotide identity between the genome obtained from isolated and genome from binning were performed by previously described method [47].

**Supplementary Results**

**Functional annotation of metagenomics assembled open reading frames**

Detection of 4-hydroxybenzaldehyde dehydrogenase (*EC 1.2.1.96*, *pchA*) / benzaldehyde dehydrogenase (*EC 1.2.1.28*, *xylC*) / salicylaldehyde dehydrogenase (*sld*) and 4-HBZ 3-monooxygenase (*EC 1.14.13.2*, *pobA*) showed that the community potentially be able to convert 4-HBD to 4-HBZ, and further transform to 3,4-dihydroxybenzoate (3,4-DHB). Two gene pathways in transformation of 3,4-DHB were detected from the community (Fig. 2). One of the pathways encodes enzymes of protocatechuate 4,5-dioxygenase (*EC 1.13.11.8*, *ligAB*), 2-hydroxy-4-carboxymuconate semialdehyde hemiacetal dehydrogenase (*EC 1.1.1.312*, *ligC*), 2-pyrone-4,6-dicarboxylate (2-PD) lactonase (*EC 3.1.1.57*, *ligI*), 4-oxalomesaconate (4-OS) tautomerase (*EC 5.3.2.8*, *galD*), 4-OS hydratase (*EC 4.2.1.83*, *ligJ*) and 4-hydroxy-4-methyl-2-oxoglutarate aldolase (*EC 4.1.3.17*, *ligK*), which transforms 3,4-DHB to 4-carboxy-2-hydroxymuconate semialdehyde (4-CHS), 2-hydroxy-2-hydropyrone-4,6-dicarboxylate (2-HHD), 2-PD, 4-OS, 4-carboxy-2-hydroxy-cis,cis-muconate (4-CHM), 4-carboxy-4-hydroxy-2-oxoadipate (4-CHO), oxaloacetate and pyruvate, finally enters the citrate cycle (4-HBZ/4-CHS-mediated pathway) (Additional file 10: Table S6).

Another pathway converts 3,4-DHB to β-CM, followed by γ-carboxymucono-lactone (γ-CL), 3-oxoadipate-enol-lactone (3-OEL), 3-oxoadipate (3-ODP), 3-oxoadipyl-CoA and succinyl-CoA, entering the citrate cycle as well (4-HBZ/β-CM-mediated pathway) (Fig. 2). This process includes genes encoding enzymes of protoascatechuate 3,4-dioxygenase (*EC 1.13.11. 3*, *pcaGH*), γ-CL hydrolase (*EC 5.5.1.2*, *pcaB*), 4-carboxymuconolactone decarboxylase (*EC 4.1.1.44*, *pcaC*), 3-oxoadipate enol-lactonase (*EC 3.1.1.24*, *pcaD*), 3-oxoadipate CoA-transferase (*EC 2.8.3.6*, *pcaIJ*), and 3-oxoadipyl-CoA thiolase (*EC 2.3.1.174*, *fadA*) (Additional file 10: Table S6).

A 4-HAP-mediated pathway was also detected from the community. The pathway catalyzes 4-HAP to 4-hydroxyphenyl acetate (4-HPAT) by 4-HAP monooxygenase (*EC 1.14.13.84*, *hapA*). 4-HPAT would then be converted to hydroquinone (HQN) by 4-HPAT hydrolase (*EC 3.1.1.2*, *hapB*). HQN dioxygenase (*EC 1.13.11.66*, *hapC*), hydroxyquinol 1,2-dioxygenase (EC 1.13.11.37, *hapD*), 4-hydroxymuconic semialdehyde dehydrogenase (*EC 1.2.1.61*, *hapE*) and maleylacetate reductase (*EC 1.3.1.32*, *hapF*) further catalyzed HQN to 4-hydroxymuconic semialdehyde (4-HMS), maleylacetate (MLL) and finally 3-oxoadipate (Fig. 2 and Additional file 10: Table S6).

**Genomes of *Sphingomonas*, *Pseudomonas* and *Pusillimonas* recovered from metagenomic datasets**

Metagenomic binning using bi-dimensional coverage plots recovered 10 genomes from the mixed community, including the genomes from the two *Sphingomonas*, the *Pseudomonas*, and the *Pusillimonas* strain (Fig. 3). The isolated *Sphingomonas* and *Pseudomonas* strain were also sequenced and assembled for obtaining genome. Annotation results revealed that all the ORFs (6210 and 5595 ORFs, respectively) as well as 16S rRNA gene sequences predicted from the assembled contigs of isolated *Sphingomonas* and the *Pseudomonas* strain were identical (in nucleotide sequence) to the ORFs predicted from binning recovered sequences of a *Sphingomonas* (Sph-2) and a *Pseudomonas* strain, repectively.

CheckM estimated the completeness of the two *Sphingomonas*, the *Pseudomonas*, and the *Pusillimonas* ranging from 96.1% to 100% (Additional file 2: Table S1). 3886, 6256, 5672, and 4050 ORFs were predicted from the four genomes respectively, where 3696, 5898, 5635 and 4025 predicted ORFs were annotated by either KEGG or NCBI-nr database fro*m* Sph-1, Sph-2*,* *Pseudomonas*, and *Pusillimonas* respectively, accounting for 95.1%, 94.3%, 99.3% and 99.4% of total predicted ORFs from those genomes, respectively. Sph-1 and Sph-2 strains shared 179 identically the same ORFs with each other, which include 7 oxygenase and oxidoreductase, 1 dehygydratase and hydratase, 3 hydorlase and 1 decarboxylase coding sequences. Totally, there is 144, 147, 95 and 179 oxygenase and oxidoreductase coding sequences detected from Sph-1, Sph-2, *Pseudomonas*, *Pusillimonas*, respectively.

**16S phylogenetic placement of the two *Sphingomonas* strains from the present study suggest the two genomes from two novel *Sphingomonas* species**

16S rRNA sequences of previous reported BPA-degrading *Sphingomonas* species and sequences with nucleotide identities ≥ 98% of Sph-1 and Sph-2 were selected as the respective sequences of *Sphingomonas* genus. Phylogenetic analysis was carried out by Mega7 with manual instruction [48]. Sph-1 and Sph-*2* from our enrichment culture formed a separate lineage with *Sphingomonas sp.* and within one strongly supported sequence cluster affiliated with *Sphingomonas daechungensis CH15-11* (Additional file 3: Figure S1), suggesting the two Sph-1 and Sph-2 probably from novel BPA-degrading species besides previously reported *Sphingomonas sp.*. Sph-1 and Sph-2 formed two separate lineages indicating the two genomes may come from two different species as well.

**Differential expression of oxidase coding genes in *Sphingomonas*, *Pseudomonas* and *Pusillimonas***

The total mapped reads to the two *Sphingomonas* species, the *Pseudomonas* species and *Pusillimonas* species accounted for 6.19%, 12.83%, 29.04%, and 11.59% of the total RNA reads from the four phases respectively. The mapped reads accounted for 64%, 73%, 85%, and 70% of total non-rRNA (including mRNA, ncRNA, etc.) in the four phases, respectively. The *Sphingomonas* shared sequences posted highest average gene expression levels. Especially in Phase III, there was 0.72% of total mapped reads for the 179 ORFs, comparing with 7.2% for 3416 ORFs in Sph-1, 1.84% for 5409 ORFs in Sph-2, 18.55% for 5477 ORFs in *Pseudomonas*, and 0.69% of 4050 ORFs in *Pusillimonas*. The shared ORFs of *Sphingomonas* species also possessed higher folder change of gene expression levels in Phase II, III and IV than the *Pseudomonas* species and *Pusillimonas* species. There were 25.3, 10.6, 13.1, 3.5, and 3 times of relative abundance shifting of gene expression of *Sphingomonas* shared ORFs, Sph-1 unique ORFs, Sph-2 unique ORFs, *Pseudomonas* ORFs and *Pusillimonas* ORFs, respectively, in Phase III of in Phase I (Additional file 4: Figure S2f).

BPA degradation and energy generation related oxidase-coding genes performed higher gene expression level (GEL) in Phase II and III in comparison with the other oxidase coding genes in the two *Sphingomonas* species. Oxidase shared by both *Sphingomonas* species, including *p450*, *pobA*, *ligAB*, *pcaG*, etc., which possibly related to BPA degradation, showed a time course of expression pattern (TCEP) of higher GEL in the Phases II and III than Phase I (Additional file 4: Figure S2a). The highest RPKM value (6746) of potential *p450* involved in BPA oxidation was ~45 times of the highest RPKM value (150) of the other 8 *p450* genes at the same phase. The potential *ferredoxin* in BPA oxidation system also expressed 24 times higher at Phase III than I, showing significant role of the *p450* system. A similar TCEP was also observed in energy generation or oxidative phosphorylation related oxidases in the two *Sphingomonas* species (Additional file 4: Figure S2b, c), such as 2-oxoacid:ferredoxin oxidoreductase (*EC 1.2.7.3*, *oor*), NAD(P)H:quinone oxidoreductase (*nour*), etc. In contrary, the other oxidases, including some of BPA possibly related genes, e.g. *hapA*, 4-hydroxyphenylpyruvate dioxidase (*EC 11.13.11.27*) and a Sph-1 strain unique sequence, *dhad,* were observed a decrease of GEL in Phases II and III, and then recovered or even increased in Phase IV.

In *Pseudomonas*, the most active oxidases in Phases II and III were *pobA* and *pcaGH* (Additional file 4: Figure S2d). Highly expression of oxidative phosphorylation related oxidases (e.g. NADH:ubiquinone oxidoreductase, etc.) had similar TCEP to *pobA* and *pcaGH*. However, GEL of hydroxyquinol 1,2-dioxidase (*EC 1.13.11.37*, *hqd*) and *hapD* showed a contrary TCEP, which decreased in Phases II and III comparing with Phase I, but recovered to similar GEL in Phase IV. Gene involved in conversion of benzoate to succinyl-CoA, catechol 1,2-dioxidase (*EC 1.13.11.1*, *ccdo*), also showed a similar TCEP with *hqd* and *hapD*. GELs of *hapA* during the Phase II, III and IV generally stay at the same level as Phase I, showing a different TCEP with *hapD*.

In the *Pusillimonas*, *pobA*, *pobB*, *pcaG*, and *pcaH* were observed highly expressed, ranging from 10-120 times in Phase II or III than I, while their activities in Phase IV returned to levels ranging from 1~4 times of GEL in Phase I, which were also similar to the oxidative phosphorylation related oxidases (Additional file 4: Figure S2e).

**Supplementary References**

47. Konstantinidis KT, Tiedje JM. Genomic insights that advance the species definition for prokaryotes. *Proc Natl Acad Sci U S A* 2005; 102: 2567-2572.

48. Kumar, S., Stecher, G., & Tamura, K. MEGA7: Molecular Evolutionary Genetics Analysis Version 7.0 for Bigger Datasets, *Mol Biol Evol* 2016; 33: 1870–1874.
