# Supplementary material for: An integrated meta-omics approach reveals substrates involved in synergistic interactions in a bisphenol A (BPA)-degrading microbial community
Source: Microbiome. 2019 Feb 6;7:16. doi: 10.1186/s40168-019-0634-5 (PMC6366072; doi:10.1186/s40168-019-0634-5)
Supplement: Supplementary file 3 — Figure S1. 16S rRNA-based phylogenetic tree of Sph-1 and Sph-2 suggests the two genomes from two different Sphinomonas species. Genomes with yellow background color suggest previously reported BPA-degrading Sphingomonas. (PDF 2537 kb) [file 40168_2019_634_MOESM3_ESM.pdf]

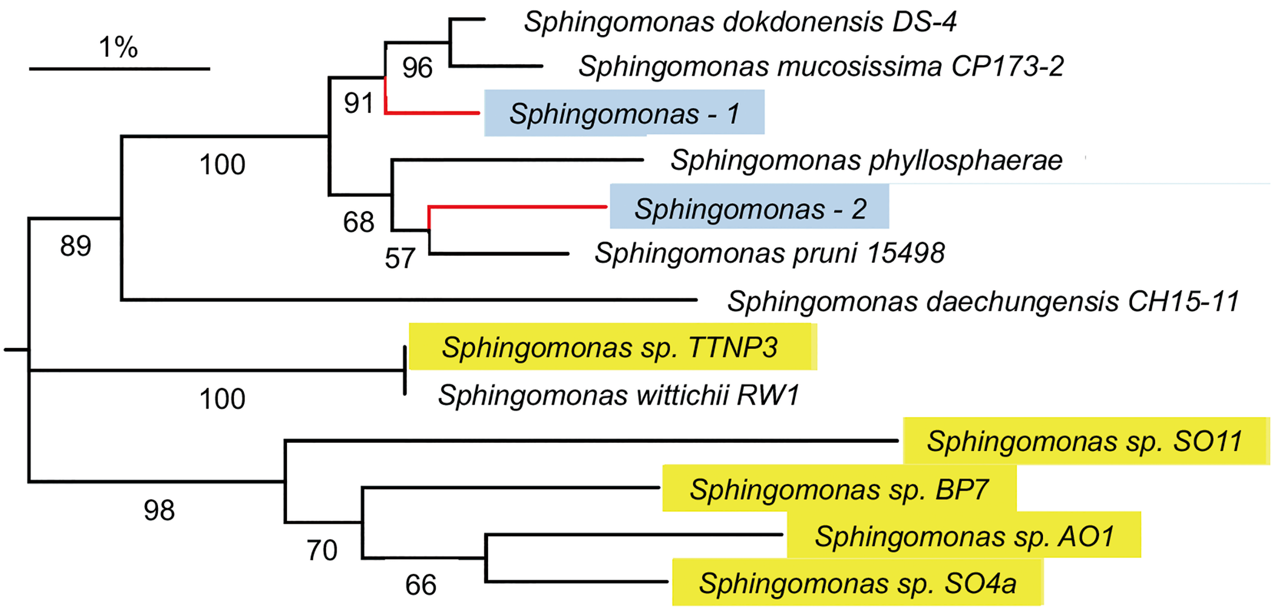

Supplementary Figure S1. 16S rRNA-based phylogenetic tree of Sph-1 and Sph-2 suggests the two genomes from two different *Sphinomonas* species. Genomes with yellow background color suggest previously reported BPA-degrading *Sphingomonas*.
