# Supplementary material for: An integrated meta-omics approach reveals substrates involved in synergistic interactions in a bisphenol A (BPA)-degrading microbial community
Source: Microbiome. 2019 Feb 6;7:16. doi: 10.1186/s40168-019-0634-5 (PMC6366072; doi:10.1186/s40168-019-0634-5)
Supplement: Supplementary file 4 — Figure S2. Heat map matrix of gene expression of oxidases (a) with identical sequences in Sph-1 and Sph-2; (b) unique to Sph-1; (c) unique to Sph-2; (d) in Pseudomonas; (e) in Pusillimonas. (f) Percentage of mapped reads in total reads of the ORFs predicted from four species and total predicted ORFs in different phases. Abbreviation indicates the enzymes coding genes possibly involved in BPA degradation process. p450, cyp450 encoding sequence (* suggest the cyp450 sequence involved in initial reaction of BPA degradation); dhbzA, protocatechuate 4,5-dioxygenase; dhbzB, protocatechuate 3,4-dioxygenase; oor, 2-oxoacid:ferredoxin oxidoreductase; hbmo, 4-hydroxybenzoate 3-monooxygenase; mhpB, 3-(2,3-dihydroxyphenyl)propionate dioxygenase; dmpB, catechol 2,3-dioxygenase; hppD, 4-hydroxyphenylpyruvate dioxygenase; dhad, 2,4′-dihydroxyacetophenone dioxygenase; qodI, quercetin 2,3-dioxygenase; co, carotenoid oxidase; hqd, hydroxyquinol 1,2-dioxygenase; hapD, hydroquinone dioxygenase; ben, benzene 1,2-dioxygenase; ccdo, catechol 1,2-dioxygenase; hapA, 4-hydroxyacetophenone monooxygenase; bphd, biphenyl 2,3-dioxygenasel; tauD, taurine dioxygenase; mqo, malate:quinone reductase (EC 1.1.5.4); nuor, NADH:ubiquinone oxidoreductase; phyH, phytanoyl-CoA dioxygenase; hgd, homogentisate 1,2-dioxygenase (EC 11.13.11.5); pdo, phthalate dioxygenase (EC 1.14.12.7); phyH, phytanoyl-CoA dioxygenase (EC 1.14.11.18); hdq, hydroxyquinol 1,2-dioxygenase. (PDF 7039 kb) [file 40168_2019_634_MOESM4_ESM.pdf]

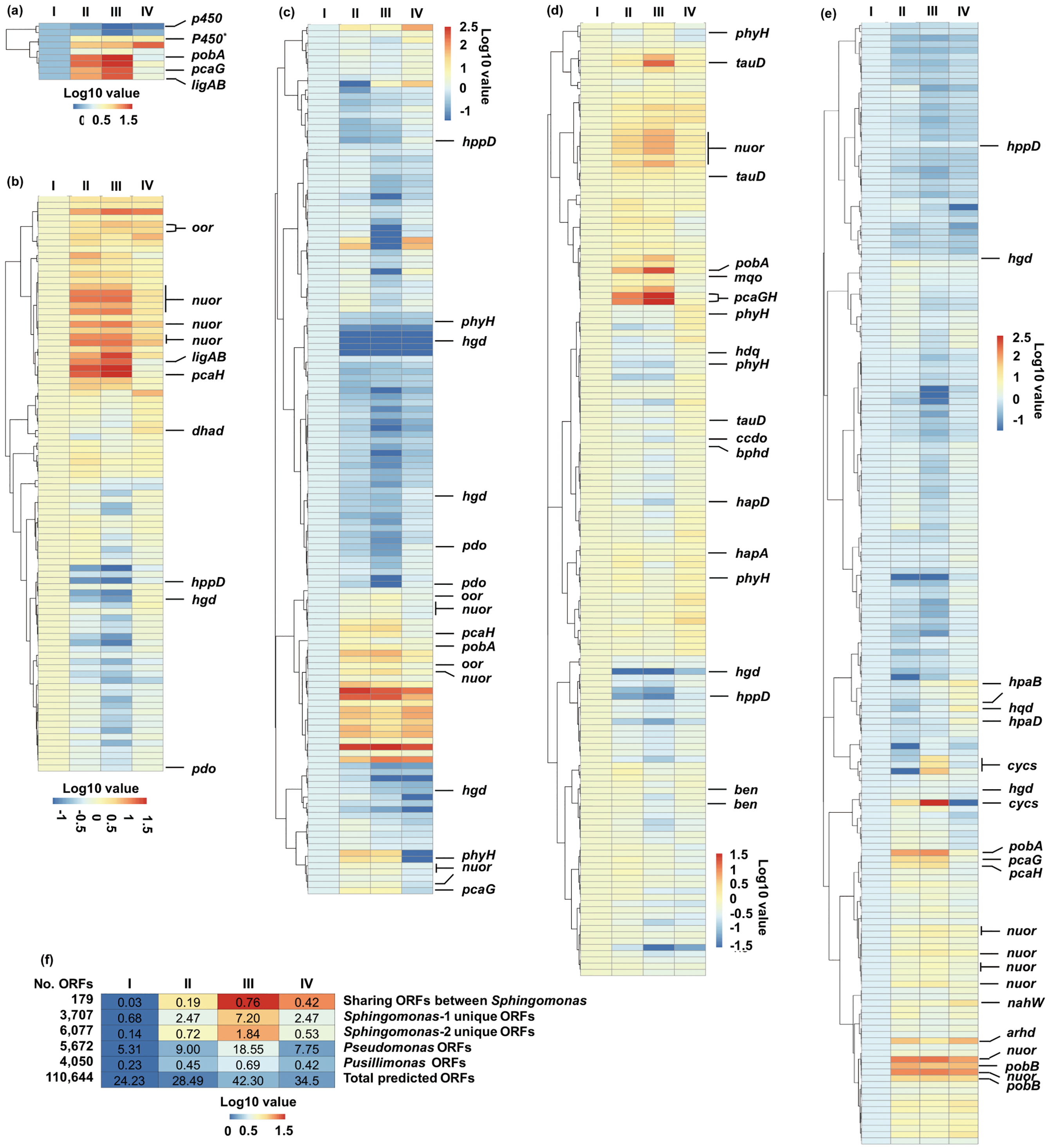

Supplementary Figure S2. Heat map matrix of gene expression of oxidases a) with identical sequences in Sph-1 and Sph-2; b). unique to Sph-1; c). unique to Sph-2; d). in *Pseudomonas*; e). in *Pusillimonas*. f). Percentage of mapped reads in total reads of the ORFs predicted from four species and total predicted ORFs in different phases. Abbreviation indicates the enzymes coding genes possibly involved in BPA degradation process. *p450*: cyp450 encoding sequence (* suggest the cyp450 sequence involved in initial reaction of BPA degradation); *dhbzA*: protocatechuate 4,5-dioxygenase; *dhbzB*: protocatechuate 3,4-dioxygenase; *oor*: 2-oxoacid:ferredoxin oxidoreductase; *hbmo*: 4-hydroxybenzoate 3-monooxygenase; *mhpB*: 3-(2,3-dihydroxyphenyl)propionate dioxygenase; *dmpB*: catechol 2,3-dioxygenase; *hppD*: 4-hydroxyphenylpyruvate dioxygenase; *dhad*: 2,4’-dihydroxyacetophenone dioxygenase; *qodI*: quercetin 2,3-dioxygenase; *co*:

carotenoid oxidase; *hqd*: hydroxyquinol 1,2-dioxygenase; *hapD*: hydroquinone dioxygenase; *ben*: benzene 1,2-dioxygenase; *ccdo*: catechol 1,2-dioxygenase; *hapA*: 4-hydroxyacetophenone monooxygenase; *bphd*: biphenyl 2,3-dioxygenasel; *tauD*: taurine dioxygenase; *mqo*: malate:quinone reductase (EC 1.1.5.4); *nuor*: NADH:ubiquinone oxidoreductase; *phyH*: phytanoyl-CoA dioxygenase; *hgd*: homogentisate 1,2-dioxygenase (*EC 11.13.11.5*); *pdo*: phthalate dioxygenase (*EC 1.14.12.7*); *phyH*: phytanoyl-CoA dioxygenase (EC 1.14.11.18); *hdq*: hydroxyquinol 1,2-dioxygenase.
