# Supplementary material for: An integrated meta-omics approach reveals substrates involved in synergistic interactions in a bisphenol A (BPA)-degrading microbial community
Source: Microbiome. 2019 Feb 6;7:16. doi: 10.1186/s40168-019-0634-5 (PMC6366072; doi:10.1186/s40168-019-0634-5)
Supplement: Supplementary file 5 — Figure S3. (a) Biodegradation of 1-BP, 4-DM and 2-BP in Sphingomonas sp. axenic culture. Biodegradation of 4-HBD, 4-HBZ, 4-HAP and 4-HPAT in Sphingomonas sp. axenic culture (b); and (c) Pseudomonas sp. axenic culture. “Sph-2” and “Pdm” indicates Sphingomonas sp. and Pseudomonas sp., respectively. Error bars indicate the standard deviation of biological triplicates. (PDF 1706 kb) [file 40168_2019_634_MOESM5_ESM.pdf]

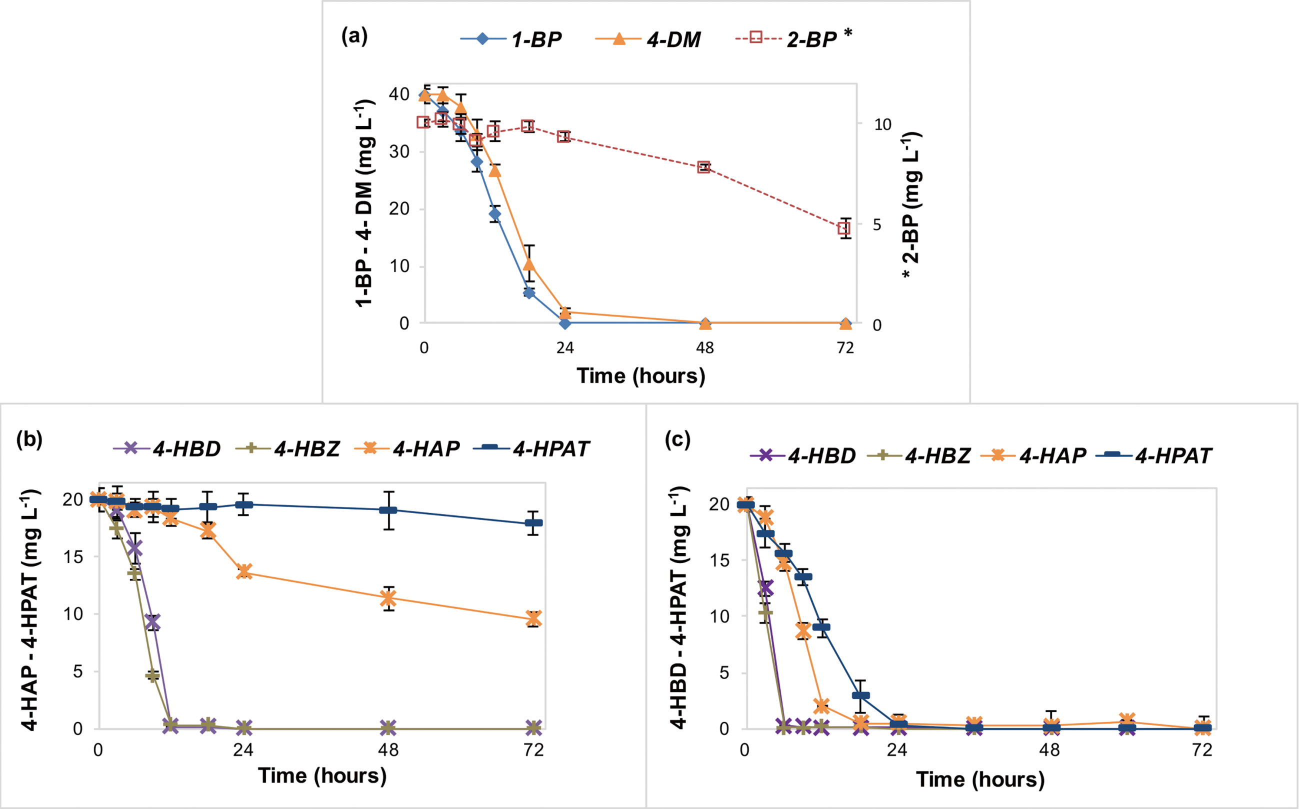

Supplementary Figure S3. (a) Biodegradation of 1-BP, 4-DM and 2-BP in *Sphingomonas sp.* axenic culture. Biodegradation of 4-HBD, 4-HBZ, 4-HAP and 4-HPAT in *Sphingomonas sp.* axenic culture (b); and (c) *Pseudomonas sp.* axenic culture. ‘Sph-2’ and ‘Pdm’ indicates *Sphingomonas sp.* and *Pseudomonas sp.*, respectively. Error bars indicate the standard deviation of biological triplicates.
